# Pediatric-onset spinocerebellar ataxia type 3 with dual *ATXN3* and *HTT* gene mutations: a case report and literature-informed hypothesis

**DOI:** 10.3389/fgene.2026.1723599

**Published:** 2026-02-10

**Authors:** Dedong Wang, Mengyao Zhou, Kang Du, Yue Wang, Kunzhi Tang, Yuanfang Duan, Mengting Shi, Haohao Wu

**Affiliations:** 1 Department of Paediatrics,Yunnan Qujing Central Hospital (Qujing First People’s Hospital), Yunnan, Qujing, China; 2 Department of Neurology, Malong District People‘s Hospital, Yunnan, Qujing, China; 3 Department of Neurology,Yunnan Qujing Central Hospital (Qujing First People’s Hospital), Yunnan, Qujing, China; 4 Department of Neurology, The Affiliated Hospital of Guizhou Medical University, Guiyang, Guizhou, China

**Keywords:** ATXN3, HTT, pediatric-onset, polyQ, SCA3

## Abstract

Spinocerebellar ataxia type 3 (SCA3) is an autosomal dominant neurodegenerative disorder caused by CAG repeat expansion in the *ATXN3* gene, typically onsetting in adults aged 30–40 years. Pediatric-onset cases are extremely rare, and concurrent CAG repeat expansions in both *ATXN3* and huntingtin (*HTT*) genes are even more exceptional. Herein, we report a 10-year-old female patient who presented with gait instability and dysarthria as initial symptoms. Diagnosis of SCA3 was confirmed by genetic and radiological evaluations. Genetic testing revealed biallelic CAG repeat lengths of 20 (normal) and 77 (expanded) in *ATXN3*, and 19 (normal) and 38 (expanded) in *HTT*. Imaging findings included mild cerebellar atrophy and bilateral tibial exostoses, consistent with her clinical phenotype. Integrated analysis of the case and a review of the literature indicated that the extreme CAG expansion in *ATXN3* (77 repeats) is the primary determinant of the remarkably early onset in this patient. The concurrent *HTT* CAG expansion may also influence the phenotype, suggesting a potential complex interaction that warrants further investigation. This case report provides a clinical example of SCA3 complicated with concurrent *ATXN3* and *HTT* mutations, offering preliminary clinical data for future large-sample studies on the correlation between these two mutations.

## Introduction

Spinocerebellar ataxias (SCAs) represent a group of neurodegenerative disorders characterized by significant phenotypic and genetic heterogeneity, encompassing numerous subtypes. The most frequent subgroup of SCAs is caused by pathogenic polyglutamine (polyQ) tract expansions. For these polyQ-related SCAs, pathological changes are primarily associated with the abnormal accumulation of mutant proteins containing expanded polyQ tracts, which lead to neuronal degeneration (Klockgether et al., 2019; Muller, 2021; Pellerin et al., 2023). Among these, SCA3 constitutes a major subtype and is inherited in an autosomal dominant manner (Bettencourt and Lima, 2011). In addition to the core manifestation of ataxia, it is frequently accompanied by various non-motor symptoms such as sleep disturbances, restless legs syndrome, cognitive impairment, depression, and sensory deficits, posing considerable challenges for clinical diagnosis and management (Hengel et al., 2023). SCA3 typically manifests in individuals aged 30–40 years with no significant gender predominance (Schöls et al., 2004). Although pediatric cases are rare, younger age at onset is associated with a poorer prognosis (Kieling et al., 2007). Pathogenic CAG repeat expansions in the *ATXN3* gene are the direct cause of SCA3, and consensually, the well-established limits of expanded alleles range from 61 to 87 repeat units (Maciel et al., 2001). To our knowledge, no prior cases of concurrent pathogenic expansions in both *ATXN3* and *HTT* genes have been reported in the literature. This article presents a case of school-aged child with SCA3 carrying concurrent mutations in the *ATXN3* and *HTT* genes, summarizing the clinical manifestations, genetic correlations, neuroimaging features, and therapeutic strategies. The aim is to contribute to the understanding of the rare co-occurrence of *ATXN3* and *HTT* mutations in SCA3, enhance precise genetic diagnosis of SCA3 is conducive to formulating individualized clinical management strategies for patients and improving their quality of life.

## Case report

**FIGURE 1:**
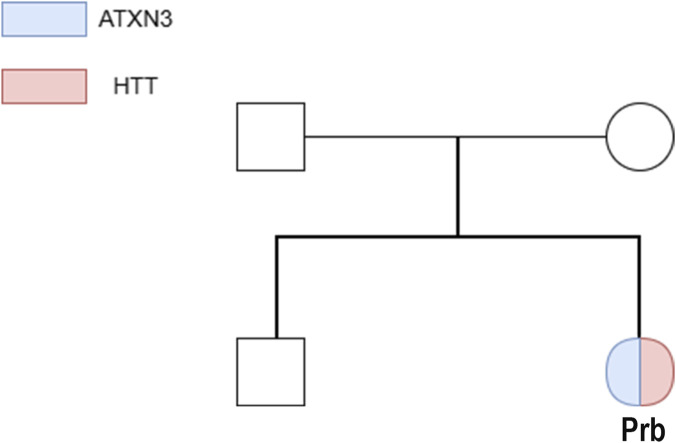
Family pedigree of *ATXN3/HTT* gene expression. Blue: *ATXN3*; Pink: *HTT*; Prb (proband) exhibits dual gene expression (colored pie chart).

A Chinese female patient began experiencing an abnormal gait at the age of 10 without obvious cause, characterized by initial contact with the toes while walking, unsteadiness, lateral swaying, and occasional falls. She was unable to walk in a straight line but could walk independently. Symptoms did not improve after calcium supplementation. Half a year later, falls became more frequent, though she remained capable of independent ambulation. One year later, she developed effortful speech, slurred articulation, and hypophonia, leading to her admission to the Department of Pediatrics. Throughout the disease course, she exhibited no limb tremors, cognitive impairment, behavioral abnormalities, visual disturbances, sleep disorders, dysphagia, or bowel/bladder dysfunction. There was no significant prior medical history. No similar hereditary conditions were reported in her family within three generations. Her father was diagnosed with “lumbar disc herniation with nerve compression” (no cerebellar/spinal cord lesions related to *SCA3*) and limping at the age of 30. He had a 20-year history of heavy physical labor (long-term bending/lifting), a well-established risk factor for degenerative lumbar disease. Moreover, he has no ataxia, dysarthria, or other neurological symptoms (core features of *SCA3*) during 10+ years of follow-up. Thus, his condition is most likely occupation-related degenerative disease, not a mild form of *SCA3*. Her mother and younger brother (8 years old) are healthy (Figure 1).

During the physical examination conducted upon the patient’s admission, she was conscious and responsive with coherent speech, though her articulation was unclear and her voice was hypophonic. Smooth pursuit movements were smooth and accurate without saccadic intrusion; saccades were initiated rapidly with normal velocity and amplitude in all directions, and no nystagmus was observed. She exhibited a wide-based gait with lateral swaying and was unable to walk in a straight line. Muscle strength was normal in all four limbs, but increased muscle tone and hyperreflexia were noted in the lower extremities. Bilateral Babinski signs were present. Finger-to-nose testing revealed dysmetria in both hands. Alternating movements were poorly coordinated. The heel-to-shin test was unsteady bilaterally. Romberg’s sign was present, and meningeal irritation signs were absent.

**FIGURE 2:**
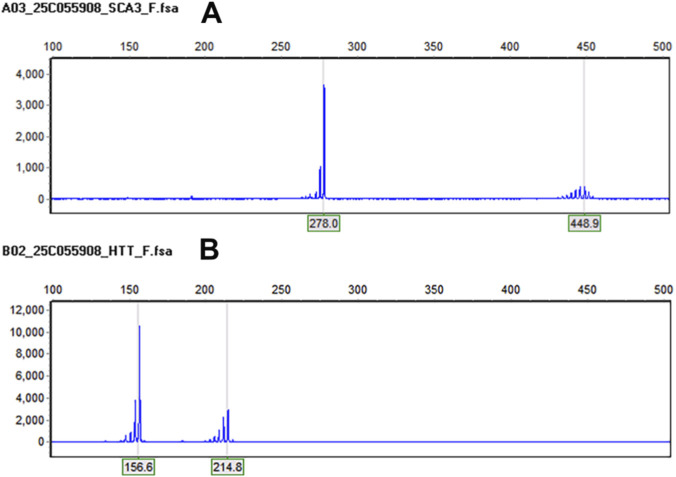
Genetic analysis: **(A)** The *ATXN3* gene (CAG) repeat numbers are 20 and 77, indicating an abnormal expansion. **(B)** The *HTT* gene (CAG) repeat numbers are 19 and 38, indicating an abnormal expansion.

**FIGURE 3:**
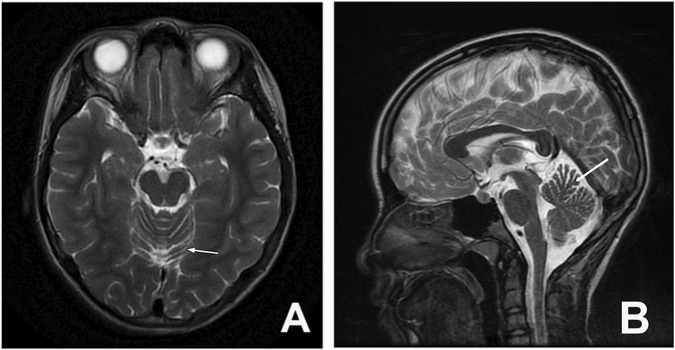
Cranial MRI: Arrow indicates mild atrophy of the anterior cerebellar lobe, no significant changes observed in the brainstem and upper cervical spinal cord (**(A)** transverse section; **(B)** sagittal section).

**FIGURE 4:**
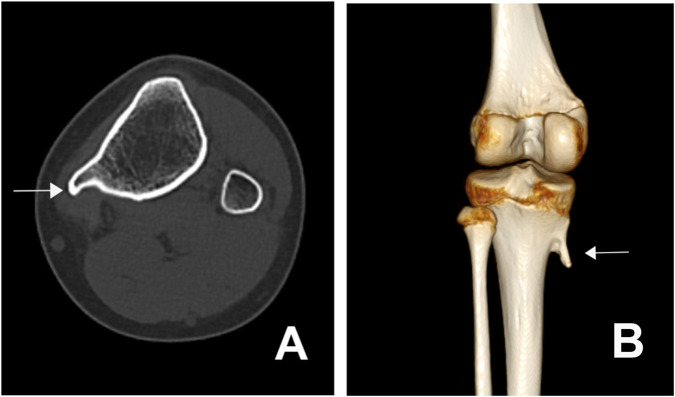
CT imaging of osteophyte formation at the knee joint. **(A)** Axial CT scan of the knee joint shows an osteophyte (white arrow) at the proximal tibia. **(B)** Sagittal 3D reconstructed CT image demonstrates the osteophyte (white arrow) projecting from the proximal tibia at the knee joint.

In the context of ancillary investigations, genetic testing revealed CAG repeat expansions in the *ATXN3* gene with repeat numbers of 20 and 77, and in the *HTT* gene with repeats of 19 and 38 (Figure 2). Epstein-Barr virus (EBV) testing was positive. MRI of the brain and cervical spinal cord showed mild atrophy of the anterior cerebellar lobe (Figure 3). CT and 3D bone imaging of the knee joints revealed bilateral exostoses in the upper tibiae (Figure 4). No significant abnormalities were detected in other laboratory tests, including complete blood count, liver and kidney function, electrolytes, blood glucose, cardiac enzymes, blood ammonia, lactate, erythrocyte sedimentation rate, serum ceruloplasmin, and thyroid function.

After integrating findings from the patient’s admission physical examination, ancillary investigations, and clinical assessment, the patient’s final diagnosis was determined to include two conditions: Spinocerebellar Ataxia Type 3 (SCA3) and Tibial exostosis.

In the sections of treatment and prognosis, the patient was treated with oral baclofen (5 mg) to reduce muscle tone. However, no significant clinical improvement was observed. The patient was subsequently discharged at the request of the guardian.

## Patients and methods

Genomic DNA was extracted from the patient’s peripheral blood leukocytes. For *ATXN3* and *HTT* genes, CAG repeat length analysis was performed using fluorescence probe PCR+capillary electrophoresis (ABI 3730xl Genetic Analyzer), combined with high-throughput sequencing (WES015: Whole Exome Sequencing V6) and Sanger sequencing validation for family members (for other candidate genes). Capillary electrophoresis, as used in our assay, can detect modest somatic mosaicism. No significant somatic expansion was observed in the patient’s blood sample. The parents declined the *ATXN3/HTT* repeat analysis due to personal concerns (psychological distress and privacy considerations).

## Discussion

SCA3 accounts for a relatively high proportion of all SCAs subtypes (Bettencourt and Lima, 2011). The core pathogenic mechanism of SCA3 is the expansion of CAG repeats in the *ATXN3* gene. This results in the production of a polyQ protein with an abnormally extended coding fragment. Meanwhile, the post-translational modification (PTM) of proteins, through phosphorylation or acetylation, causes abnormal aggregation of the polyQ protein and affects its intracellular solubility. This promotes the abnormal accumulation of the polyQ protein in specific neuronal subpopulations such as cerebellar Purkinje cells, ultimately leading to progressive degeneration of neurons (Puranam et al., 2006; Wan et al., 2018; Xiang et al., 2018). When lesions affect the neural pathways in key motor regulatory regions such as the cerebellum, brainstem, and spinal cord, typical symptoms of ataxia—including abnormal gait—emerge. If the pathology involves the cerebellar vermis or brainstem, dysarthria may further develop (Sambataro and Pennuto, 2012). The onset and progression of symptoms in this case are consistent with the core clinical manifestations of SCA3. Genetic testing confirming a mutation in the *ATXN3* gene provides clear evidence supporting the diagnosis of SCA3.

However, what is particularly notable in this case is the unusually early age of onset. As mentioned previously, the average onset of SCA3 typically occurs in early adulthood and is rarely observed in children. When the disease manifests at a very young age (e.g., before 10–15 years), patients often experience rapidly progressive brainstem and cerebellar failure, leading to premature death (Guo et al., 2025). Multiple studies on the age of onset and clinical features of SCA3 have revealed that a higher number of CAG repeats in the *ATXN3* gene correlates with an earlier disease onset, greater polyglutamine (polyQ) expansion, more prominent protein misfolding and aggregation, and more severe clinical symptoms (Li et al., 2020; Tang et al., 2000; Tezenas et al., 2023). Donis et al. (Da Silva Carvalho et al., 2016) shows a case with approximately 77 *ATXN3* repeats and an onset age around 20 years. In this case, the patient’s *ATXN3* gene exhibited CAG repeat numbers of 20 and 77. The high repeat number is one of the key factors contributing to the earlier onset compared to the average age of SCA3 presentation. Additionally, the abnormal CAG expansion in the *HTT* gene observed in this patient warrants attention. Although no direct association between *HTT* and SCA3 has been established previously, instead, *HTT* is mostly recognized as the pathogenic gene for Huntington’s disease (HD). It is noteworthy that abnormal CAG expansions in *HTT* also show an inverse correlation with the age of onset in Huntington’s disease (typically >36 repeats, and >60 in juvenile cases). This suggests that polyQ-related disorders may share common underlying pathogenic mechanisms (Ajitkumar et al., 2025; Chakraborty et al., 2024). However, the patient did not exhibit typical clinical features of Huntington’s disease (Stoker et al., 2022), and it is considered that the CAG repeat numbers (19 and 38) in the *HTT* gene in this age group are likely insufficient to induce the onset of Huntington’s disease (Lee et al., 2012). But it has been reported to modify phenotypes of other polyglutamine disorders (Hong et al., 2024). We propose that the HTT low penetrance range may accelerate the onset of SCA3 in our patient, even with fewer *ATXN3* repeats, aligning with emerging evidence of cross-pathway interactions between polyglutamine-expanded proteins. Furthermore, basic research focusing on the relationship between *HTT* and SCA3 has suggested that huntingtin-associated protein 1 (*HAP1*) may modulate the physiological function of *ATXN3*, thereby contributing to the pathogenesis and progression of SCA3 (Gao et al., 2019). Notably, the coexistence of *ATXN3* pathogenic expansion and *HTT* low penetrance range in this patient is an exceptionally rare event, and no causal link between the two expansions has been established to date. To date, only a handful of case reports have documented this dual mutation scenario, and their phenotypic presentations exhibit striking heterogeneity. Beyond *ATXN3-HTT* dual mutations, reports of other combined triplet elongations in polyQ disorders also provide critical insights into cross-gene interactions. Tezenas et al. demonstrated that the co-occurrence of *ATXN3* pathogenic expansion and *ATXN1* CAG repeat elongation exacerbated protein aggregation and neurodegeneration in SCA3 patients, leading to earlier onset and more severe phenotypes (Tezenas Du Montcel et al., 2014). Notably, the CAG expansion in the HTT gene may have delayed the onset of SCA3 in this case, pending confirmation of the underlying mechanism via further locus expansion studies (Tezenas Du Montcel et al., 2014). A high CAG repeat length in the *ATXN3* gene was identified as the main driver of early disease onset in this patient, while the potential influence of concurrent *HTT* intermediate expansions requires validation in large-sample studies. This rare pediatric case with both pathogenic *ATXN3* CAG expansion and intermediate *HTT* CAG expansion offers important clinical insights into the phenotypic presentation and potential genetic interactions of dual polyQ-expanded diseases. We propose that the accumulation of more genetic and clinical data will further elucidate the pathogenic interplay among *ATXN3, HTT*, and SCA3, enabling more comprehensive characterization of the disease.

Consistent with other findings (Zhou et al., 1997), our patient exhibited core clinical manifestations typical of early-onset SCA3, including progressive cerebellar ataxia, dysarthria, and oculomotor dysfunction, which were tightly correlated with an exceptionally high ATXN3 CAG repeat length. However, a critical distinction emerged: unlike the majority of juvenile-onset cases described in the reference, which presented with isolated ATXN3 pathogenic expansions and unmodulated disease progression, our patient harbored concurrent intermediate CAG expansions in the HTT gene, a feature that may have contributed to the relatively delayed disease onset despite the high ATXN3 repeat burden.

Neuroimaging alterations in SCA3 can involve extensive brain structures, with brainstem and cerebellar atrophy being particularly prominent. Studies have shown an inverse correlation between CAG repeat length in the *ATXN3* gene and volumes of the brainstem, cerebellum, and basal ganglia. The degree of atrophy also correlates positively with symptom severity and may contribute to a higher burden of non-motor symptoms, further elucidating the association between early onset and severe clinical manifestations in SCA3 (Wan et al., 2020; Yap et al., 2022). However, in this pediatric case, brain MRI revealed only mild atrophy of the anterior cerebellar lobe, and the clinical presentation was limited to typical motor symptoms of SCA3 (the tibial exostoses were considered to be either stress-induced adaptive bone hyperplasia due to long-term abnormal gait or a primary bone developmental anomaly, rather than a non-motor symptom). These findings are not entirely consistent with the conclusions of the aforementioned studies, which may be attributed to the relatively short disease duration and potentially explained by a complex interaction associated with the *HTT* mutation. Studies have suggested that a larger CAG repeat in the *HTT* gene may enhance its binding to mutant protein fragments, preventing their interference with other functional proteins and thereby reducing proteotoxicity and delaying neural damage (Tezenas Du Montcel et al., 2014). Consequently, SCA3 patients with concurrent *HTT* mutations may exhibit slower progression of clinical symptoms and less severe structural brain impairment.

Currently, there is no curative treatment for SCA3. The primary management strategies focus on early symptomatic intervention, maintaining functional independence, and preventing or treating complications to improve quality of life and slow disease progression—particularly in young patients. Therefore, for individuals presenting with ataxia at an atypical age, prompt genetic testing to clarify the etiology and initiate early treatment is essential. Furthermore, genetic research has opened new avenues for SCA3 therapy. In recent years, gene-targeted therapies have become an active area of investigation, including approaches such as anti-*ATXN3* antisense oligonucleotides (ASOs) and *in vivo* self-assembling siRNA therapeutics. However, these treatments remain in the experimental stage (Schuster et al., 2024). Thus, genetic studies hold significant value for understanding the pathology, improving clinical diagnosis and management, and interpreting neuroimaging changes in SCA3.

## Conclusion

This study reports a rare case of school-aged SCA3 with dual mutations in the *ATXN3* and *HTT* genes. The mutation in *ATXN3* is a well-established causative factor and represents a key molecular genetic determinant contributing to early onset, severe manifestations, early mortality, and significant neuroimaging abnormalities. Unusually, the co-occurrence of an abnormal CAG expansion in the *HTT* gene may interact synergistically with *ATXN3* in the pathogenesis of SCA3, yet its effect on age of onset appears to be delayed rather than accelerated. Moreover, the increased CAG repeat length in *HTT* may exert a neuroprotective effect in SCA3, offering a theoretical explanation for the observed milder clinical and radiological severity in this patient despite the high CAG repeat number in *ATXN3*. However, the interpretation of our findings must be tempered by the relatively short duration of follow-up (1 year). While the clinical parameters in our cohort appeared stable during this period, this is insufficient to conclusively determine a delayed disease course. A more definitive assessment would require longitudinal studies with extended follow-up periods. Furthermore, our observations align with the heterogeneous progression rates reported in the literature on juvenile-onset SCA3 (Donis et al., 2016). Some studies have described a more aggressive trajectory in very young onset cases, underscoring the wide phenotypic variability of this disorder (Moura et al., 2024). Currently, there is no curative treatment for SCA3, and the prognosis remains poor. Gene-targeted therapy may represent a promising avenue for precise intervention in SCA3 in the future. We recognize that genetic counseling plays a crucial role in clarifying inheritance patterns more explicitly, thereby optimizing risk stratification for at-risk relatives.

## Data Availability

The datasets presented in this study can be found in online repositories. The names of the repository/repositories and accession number(s) can be found in the article/supplementary material.
